# TGF-β-Enriched Exosomes from Acute Myeloid Leukemia Activate Smad2/3–MMP2 and ERK1/2 Signaling to Promote Leukemic Cell Proliferation, Migration, and Immune Modulation

**DOI:** 10.3390/cimb47090690

**Published:** 2025-08-27

**Authors:** Jie Jia

**Affiliations:** School of Chemical Engineering, Ocean and Life Sciences, Dalian University of Technology, Panjin 124221, China; jiajie1027@mail.dlut.edu.cn

**Keywords:** AML, exosomes, TGF-β, proliferation, migration, immune modulation

## Abstract

Exosomes are extracellular vesicles secreted by all cell types, transporting nucleic acids, proteins, lipids, and metabolites. They are known to influence tumor biology by modulating cellular proliferation, invasion, and apoptosis. In acute myeloid leukemia (AML), the precise functions of exosomes remain incompletely characterized. Here, we present an integrated multi-omics study combining single-cell RNA sequencing (scRNA-seq) of bone marrow aspirates from AML patients and healthy donors with transcriptomic profiling of purified exosomes. This approach uniquely allowed us to link cellular transcriptional states with exosome content and function. We discovered a significant upregulation of exosome-related transcriptional activity in AML cells. Purified AML exosomes showed enhanced translational, transcriptional, and metabolic activity compared to those from healthy donors. Notably, these exosomes were highly enriched in transforming growth factor-β (TGF-β), a key regulator of tumor progression. Functional assays confirmed that AML-derived exosomes promote leukemic cell proliferation and migration. Mechanistically, these effects are mediated via activation of the Smad2/3–MMP2 and ERK1/2 signaling pathways. Furthermore, cell–cell interaction analysis revealed that AML exosomes reshape the bone marrow immune microenvironment by upregulating multiple immunoregulatory genes and pathways, revealing a novel immunomodulatory role. This study provides the first integrative demonstration that TGF-β–enriched exosomes actively drive AML progression through combined enhancement of leukemic aggressiveness and immune microenvironment remodeling. Our findings highlight exosomes and their signaling cascades as promising therapeutic targets, offering new avenues for innovative AML treatments.

## 1. Introduction

Acute myeloid leukemia (AML) ranks among the most frequently occurring malignant tumors, noted for the unregulated growth of myeloid cells. The likelihood of developing AML rises with age, with older patients typically experiencing poorer outcomes compared to younger counterparts. Current treatment modalities for AML—such as chemotherapy, radiation, and stem cell transplantation—offer limited success in achieving full remission or long-term disease-free survival [1,2]. Therefore, investigating the fundamental mechanisms that drive AML progression is crucial for enhancing patient outcomes.

Recently, the role of exosomes in cancer has gained substantial attention. Exosomes are lipid bilayer vesicles, approximately 30–200 nm in diameter, originating from the endosomal pathway. They develop within intracellular multivesicular bodies and are released into the extracellular space through exocytosis [3]. Evidence increasingly supports the involvement of tumor-derived exosomes in intercellular communication among cancer cells, as they facilitate the transfer of oncogenic molecules, including proteins, DNA, mRNA, microRNA, and lipids, thereby impacting processes like aberrant angiogenesis, tumor progression, metastasis, and resistance to therapies [4,5,6].

In patients with newly diagnosed, untreated AML, plasma exosome concentrations are significantly elevated compared to healthy individuals. These exosomes carry a variety of bioactive molecules, including membrane-bound transforming growth factor beta 1 (TGF-β1), which plays a pivotal role in immune modulation [7]. TGF-β1 is a multifunctional cytokine with well-documented dual roles in tumor biology. In the early stages of cancer development, it often acts as a tumor suppressor by inhibiting cell proliferation and inducing apoptosis [8,9]. It is demonstrated that exosomes derived from AML patients’ plasma suppress natural killer (NK) cell cytotoxicity by downregulating the activating receptor NKG2D, thereby impairing NK cell-mediated tumor surveillance [10,11]. Further research by Tohumeken et al. revealed that AML-derived exosomes can reprogram monocytes into myeloid-derived suppressor cells (MDSCs) via the TLR2/Akt/mTOR signaling pathway. These MDSCs exhibit an immunosuppressive phenotype, characterized by the expression of indoleamine 2,3-dioxygenase (IDO), S100A8/9, and C/EBPβ, and a glycolytic metabolic shift that enhances their suppressive functions [12]. However, as the tumor progresses, TGF-β1 frequently switches roles to promote tumor growth, invasion, metastasis, and immune evasion [8,13]. TGF-β1 is known to initiate a canonical signaling cascade involving Smad2/3 phosphorylation and subsequent nuclear translocation, as well as non-canonical activation of the ERK1/2 pathway [14,15]. These cascades are well-characterized in the context of epithelial–mesenchymal transition (EMT) in solid tumors, where they promote cell migration, invasion, and extracellular matrix remodeling [16]. While EMT is not a classical process in hematological malignancies, similar functional outputs—such as increased motility, proliferation, and matrix degradation—are relevant to leukemia progression and dissemination [17,18].

Despite the central role of TGF-β–Smad/ERK signaling in cancer biology, its exosome-mediated activation in AML has not been well defined. This study represents an investigation into the impact and core molecular mechanisms of AML-exos in AML progression. We discovered that AML-exos promote AML cell proliferation and metastasis through the TGF-β signaling pathway. These findings enhance our comprehension of the interactions between AML-exos and AML cells, providing a foundational perspective for further studies on AML pathogenesis. Collectively, these findings underscore the multifaceted role of AML-derived exosomes in modulating the immune microenvironment. A comprehensive understanding of the molecular mechanisms underlying exosome-mediated immune modulation in AML is crucial for developing targeted therapeutic strategies aimed at restoring immune surveillance and improving patient outcomes.

## 2. Materials and Methods

### 2.1. Cell Culture

Thp-1, a human monocytic leukemia cell line, was sourced from ATCC (catalog no. TIB-202) and cultured under standard conditions. Cells were maintained in high-glucose DMEM (Hyclone, Logan, UT, USA) supplemented with 10% fet al bovine serum (FBS) that had been depleted of exosomes, along with 100 U/mL penicillin and 100 μg/mL streptomycin. To eliminate contaminating exosomes, FBS was ultracentrifuged at 100,000× *g* at 4 °C overnight prior to use. Cultures were incubated at 37 °C in a humidified environment containing 5% CO_2_. Only early-passage cells (passages 3 to 6) were utilized in all experimental assays to ensure consistency and minimize phenotypic drift.

### 2.2. Isolation of Exosomes

Exosomes were extracted from cell culture media using a standard differential centrifugation method as previously described [19]. To isolate exosomes, conditioned medium was harvested from Thp-1 cell cultures after 72 h of incubation. An initial low-speed spin at 300× *g* for 10 min was performed to remove intact cells, followed by centrifugation at 2000× *g* for 10 min to eliminate dead cells and residual debris. The clarified supernatant then underwent centrifugation at 10,000× *g* for 30 min to remove larger vesicles and apoptotic bodies. The resulting supernatant was transferred into clean tubes and ultracentrifuged at 100,000× *g* for 70 min at 4 °C to pellet small extracellular vesicles. The pellet was washed in phosphate-buffered saline (PBS), passed through a 0.22 μm filter to eliminate residual impurities, and subjected to a second round of ultracentrifugation under the same conditions. Final exosome pellets were resuspended in PBS and stored at –80 °C until use. Total protein concentration was measured using a BCA assay kit (Beyotime, Cat# P0012S, Shanghai, China).

### 2.3. Transmission Electron Microscopy

Isolated exosomes were fixed in 2.5% glutaraldehyde for 5 min, then placed on formvar carbon-coated 400-mesh copper grids. After negative staining with 2% phosphotungstic acid for 2 min, grids were air-dried, and exosome morphology and size were imaged using a transmission electron microscope (Tecnai G2F30 STWIN, FEI, Hillsboro, OR, USA) operating at 100 keV.

### 2.4. Dynamic Light Scattering Analysis

Exosome size and distribution were assessed with a Malvern Zetasizer Nano ZS (Nano ZS90, Malvern, Malvern, UK) equipped with a 532 nm laser. Exosome samples were resuspended in PBS, vortexed for 1 min, and loaded into the measurement chamber at ambient temperature. Measurements were conducted automatically and analyzed using Zetasizer software (version 8.02).

### 2.5. Exosome Labeling and Tracing

Exosomes were fluorescently labeled using the lipophilic dye DIO in accordance with the manufacturer’s instructions. After labeling, the exosomes were incubated with Thp-1 cells at 37 °C for 24 h to allow for uptake. Following incubation, cells were rinsed thoroughly with ice-cold PBS to remove unbound exosomes, then fixed in 4% paraformaldehyde for 15 min. Nuclear staining was performed using DAPI for 5 min at room temperature. The cellular localization of internalized exosomes was visualized using a laser scanning confocal microscope (Leica DMI6000CS, Leica Microsystems, Wetzlar, Germany).

### 2.6. Cell Proliferation Test

To evaluate the effects of exosomes on cell viability, Thp-1 cells were plated at a density of 1 × 10^4^ cells per well in 96-well round-bottom plates (Costar, Cat. #3799, Corning, Corning, NY, USA). Cells were exposed to varying concentrations of exosomes (0–30 μg/mL) and cultured for 120 h. Bright-field images were acquired at 50× magnification using a Leica DM14000B optical microscope (Leica DMI6000CS, Leica Microsystems, Wetzlar, Germany) to document morphological changes. Cell viability was assessed using the Cell Counting Kit-8 (CCK-8, APExBIO, Irving, TX, USA, Cat. #K1018), following the manufacturer’s instructions. Absorbance was measured at 450 nm with a microplate reader (Infinite M Nano+, Tecan, Männedorf, Switzerland), and results were normalized to the untreated control group to determine relative viability.

For growth assessment, Thp-1 cells pre-treated with or without ITD-1 (TGF-β receptor inhibitor; SF7899, Beyotime, 5 μM) for 24 h were seeded into 96-well plates (1 × 10^4^ cells/well), then treated with 30 μg/mL of exosomes or PBS as control. Images were taken at 50× magnification, and cell counts were determined in triplicates on day 2.

### 2.7. Cell Migration Assay

For transwell migration assays, Thp-1 cells were pre-treated with 5 μM ITD1 or vehicle control for 24 h. A total of 2 × 10^5^ cells suspended in serum-free medium were seeded into the upper chamber of Transwell inserts with 5 μm pore membranes (Costar, Cat. #3421, Corning). The lower chamber was filled with serum-free medium containing 30 μg/mL of exosomes, while control wells received exosome-free medium. After incubation at 37 °C for 18 h, cells that had migrated through the membrane to the lower chamber were collected and counted manually using a hemocytometer.

### 2.8. Western Blotting

Western blotting was performed following previously established methods [20,21]. Protein concentrations of polyacrylamide were set between 10 and 15%, depending on the molecular weight of the target protein. Primary antibodies used included Alix (ab186429, Abcam, Cambridge, UK), CD63 (UR52301-3, Umibio, Shanghai, China), TSG101 (UR52301-4, Umibio), TGF-β (bs-4538R, Bioss, Bejing, China), phospho-ERK1/2 (Thr202/Tyr204) (bs-3016R, Bioss), ERK1/2 (AF1051, Beyotime), phospho-Smad2(S465/467)/Smad3(S423/425) (AP0548, ABclonal, Wuhan, China), Smad2/3 (AF8001, Beyotime), MMP2 (AF1420, Beyotime), Bax (50599-2-lg, Proteintech, Wuhan, China), Bcl-2 (12789-1-AP, Proteintech), and β-actin (AF5001, Beyotime), incubated overnight at 4 °C. After washing, blots were treated with anti-rabbit IgG-HRP (A0208, Beyotime), and anti-mouse IgG-HRP (A0216, Beyotime) (0.04 μg/mL) for 60 min. Immunoreactive bands were ultimately visualized using ECL reagents (P0018S, Beyotime) and detected with the Imaging System (FluorChem HD2, ProteinSimple, San Jose, CA, USA). In some experiments, the blot was reprobed with another antibody after 30 min at 50 °C in 62.5 mM Tris-HCl (pH 6.7), 100 mM 2-mercaptoethanol, and 2% SDS buffer.

### 2.9. Dataset Information of Transcriptomics Analysis

Bulk RNA sequencing data derived from exosomes isolated from the bone marrow serum of 6 patients diagnosed with AML and 4 healthy donors were retrieved from the Gene Expression Omnibus (GEO) under the accession number GSE285301 [19]. The single-cell RNA-seq data from bone marrow aspirates of 4 healthy control donors and 6 AML patients at diagnosis were obtained from GSE116256 [22].

### 2.10. Gene Set Enrichment Analysis

Gene set enrichment analysis (GSEA) was carried out to identify altered biological process difference between AML patients and healthy donors, utilizing the tool provided by the GSEA-MSigDB platform (https://www.gsea-msigdb.org/gsea/index.jsp, accessed on 20 August 2025) [23,24]. The Gene Ontology-Biological Process database was used as reference. Pathways with a normalized enrichment score (NES) exceeding 1 and a *p*-value under 0.05 were considered significantly enriched.

### 2.11. Single-Cell RNA-Seq Processing

Single-cell RNA-seq data from GSE116256 were downloaded as a Seurat object in RDS format [25]. Quality control excluded cells with fewer than 200 or more than 7000 detected genes, or over 5% mitochondrial gene expression. The data were normalized, scaled, and highly variable genes identified using Seurat. PCA was performed on the top 2000 variable genes, followed by clustering with a resolution of 0.5 based on the first 20 PCs. Marker genes for each cluster were detected to assist cell type annotation. UMAP was performed to visualize cells according to different cell types, disease states, and individual samples.

### 2.12. Cell–Cell Interaction Analysis

Cell–cell interaction analysis was performed using the CellChat R package (version 2.1.0) [26]. The Seurat object containing annotated cell types was used as input. The createCellChat function was applied to initialize the CellChat object, specifying the cell identity as the grouping variable. The ligand–receptor interaction database was set to the built-in CellChatDB for human. Overexpressed genes and interactions were identified with default thresholds. Cell–cell communication probabilities were computed using the computeCommunProb function with standard parameters. Significant interactions were inferred after filtering using permutation tests (computeCommunProbPathway and filterCommunication). Network visualization and analysis, including interaction strength and signaling pathway contributions, were generated with the corresponding plotting functions provided by CellChat.

### 2.13. Statistical Analysis

All statistical analyses were carried out using SPSS software (version 25.0). Experimental data are reported as mean values with standard deviation (±s.d.), based on at least three biologically independent replicates. Data visualization and figure preparation were performed using GraphPad Prism 8.0. For comparisons involving two experimental groups, statistical significance was assessed using an unpaired Student’s *t*-test. When analyzing three or more groups, one-way analysis of variance (ANOVA) was employed. A *p*-value of less than 0.05 was considered indicative of statistical significance.

## 3. Results

### 3.1. Single-Cell Transcriptomic Profiling Reveals Disrupted Hematopoiesis and Enhanced Exosome Activity in AML Bone Marrow

**Figure 1:**
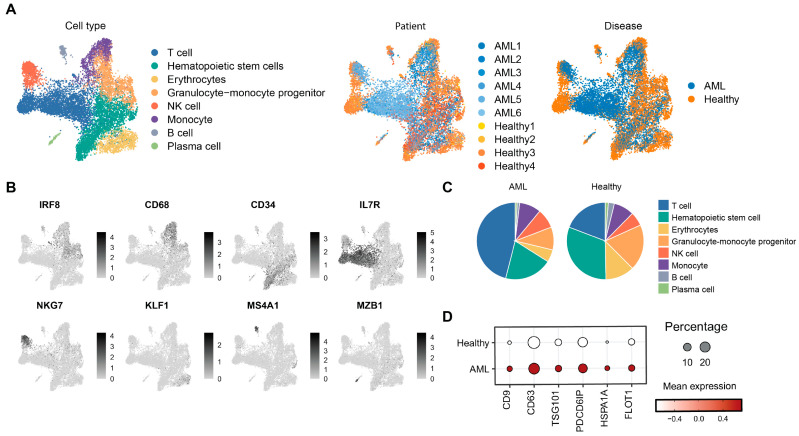
Single-cell analysis of bone marrow aspirate from AML patients and healthy donors. (**A**) UMAP visualization of single-cell RNA-seq data, grouped by cell type, disease status, and individual patients. (**B**) FeaturePlot showing expression of marker-specific genes. (**C**) Distribution of cell types in AML and healthy donors. (**D**) Expression of exosome markers in AML and healthy donors.

We performed single-cell RNA sequencing analysis on bone marrow samples from six AML patients and four healthy donors. After rigorous quality control and filtering, approximately 6151 cells from AML patients and 5421 cells from healthy donors were retained for subsequent analysis (Figure 1A). By leveraging canonical cell type-specific marker genes, we identified distinct populations including T cells, hematopoietic stem cells (HSCs), erythrocytes, granulocyte–monocyte progenitors, natural killer (NK) cells, monocytes, B cells, and plasma cells (Figure 1B) [22]. A quantitative comparison of cellular composition revealed a pronounced increase in the proportion of T cells in AML samples, alongside a substantial reduction in HSCs, erythrocytes, and granulocyte–monocyte progenitors (Figure 1C). These findings indicated the disruption of normal hematopoiesis in AML, characterized by ineffective differentiation and the accumulation of immature myeloid blasts that crowd out healthy progenitor populations. The decrease in erythrocytes aligns with the anemia commonly observed in AML patients [27], while the reduced granulocyte–monocyte progenitors indicate impaired myeloid lineage development [28]. Interestingly, the expression levels of canonical exosome markers were significantly elevated in AML cells compared to controls (Figure 1D). This suggests enhanced exosome biogenesis and secretion, which may facilitate intercellular communication within the leukemic bone marrow microenvironment.

### 3.2. Exosomal Transcriptomic Signatures Reveal Enhanced Ribosome Biogenesis and Metabolic Reprogramming in AML Patients

**Figure 2:**
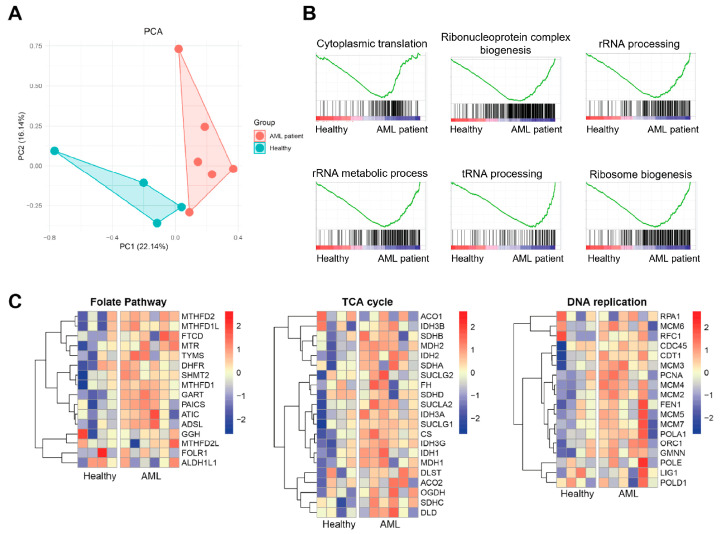
Transcriptomic comparison of bone marrow derived-exosomes between AML patients and healthy donors. (**A**) PCA plot comparing AML patients and healthy donors. (**B**) GSEA of key rRNA, tRNA, and ribosome-related pathways upregulated in AML patients. (**C**) Heatmap of upregulated metabolic pathways in AML patients and healthy donors.

To explore the potential contribution of exosomes to AML pathogenesis, we re-analyzed bulk RNA sequencing of bone marrow serum-derived exosomes from six patients with AML and four healthy controls. Principal component analysis (PCA) revealed a clear separation between the AML and control groups, suggesting substantial alterations in the transcriptomic profiles of AML-derived exosomes (Figure 2A). Unbiased gene set enrichment analysis (GSEA) demonstrated significant enrichment of ribosome-related pathways, including rRNA processing, tRNA metabolism, and ribosome biogenesis, in the AML group (Figure 2B). This suggests that exosomes released by AML cells are enriched in components associated with elevated translational capacity and biosynthetic activity, which are related to the high proliferative rate and protein production demands of leukemic blasts. Further analysis revealed upregulation of metabolic pathways such as the folate biosynthesis pathway, tricarboxylic acid (TCA) cycle, and DNA replication, indicating that exosomal contents reflect the hyperactive metabolic state of AML cells (Figure 2C). These metabolic shifts are consistent with the known metabolic reprogramming in leukemia, which supports rapid cell division, survival under stress, and resistance to therapy [29]. Altogether, these findings suggest that AML-derived exosomes carry molecular signatures indicative of leukemic pathophysiology, including enhanced ribosomal and metabolic activity, and may play an active role in reshaping the bone marrow microenvironment to promote leukemic cell survival, immune evasion, and disease progression.

### 3.3. AML-Exos Affect Thp-1 Cell Proliferation and Migration

**Figure 3:**
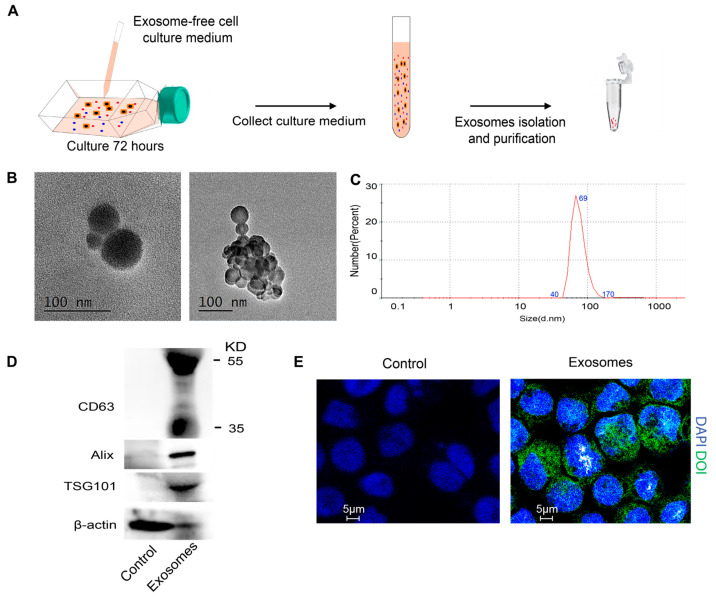
Isolation and identification of AML-exos. (**A**) Flow chart for the separation of exosomes from cell culture medium. (**B**) Morphologies of exosomes were observed via transmission electron micrograph. Scale bar = 100 nm. (**C**) Size distribution of exosomes was measured by dynamic light scattering analysis, peaking at 69 nm. (**D**) The expression of tetraspanin CD63, ALG-2-interacting protein X (Alix), and tumor susceptibility gene 101 (TSG101) in the Thp-1 cell lysate (Control), or AML-exos (Exosomes) were detected by Western blotting. (**E**) Confocal microscopy images of Thp-1 cells incubated with exosomes for 24 h. The nucleus was stained with DAPI (blue), and exosomes were stained with DIO (green). Scale bar = 5 μm.

To further investigate the biological properties and potential functional impact of AML-derived exosomes, we isolated exosomes from the culture medium of Thp-1 cells, a human monocytic AML cell line representative of the M5 subtype. Thp-1 cells were cultured in exosome-depleted medium for 72 h, and exosomes (AML-exos) were collected by differential ultracentrifugation (Figure 3A). The vesicles demonstrated typical exosomal traits, with sizes ranging between 42 and 94 nm, as confirmed by transmission electron microscopy (TEM) (Figure 3B). Dynamic light scattering (DLS) analysis indicated that AML-exos exhibited a diameter distribution from 40 to 170 nm (Figure 3C). Additionally, Western blotting identified exosome markers CD63, Alix, and TSG101 within AML-exos (Figure 3D). To examine whether AML-exos could be taken up by Thp-1 cells and influence cellular functions, Thp-1 cells were incubated with DIO-labeled AML-exos for 24 h. Confocal microscopy showed efficient uptake of AML-exos by Thp-1 cells (Figure 3E). In summary, we successfully isolated and characterized exosomes derived from Thp-1 AML cells, confirming their typical exosomal features and demonstrating efficient uptake by AML cells.

**Figure 4:**
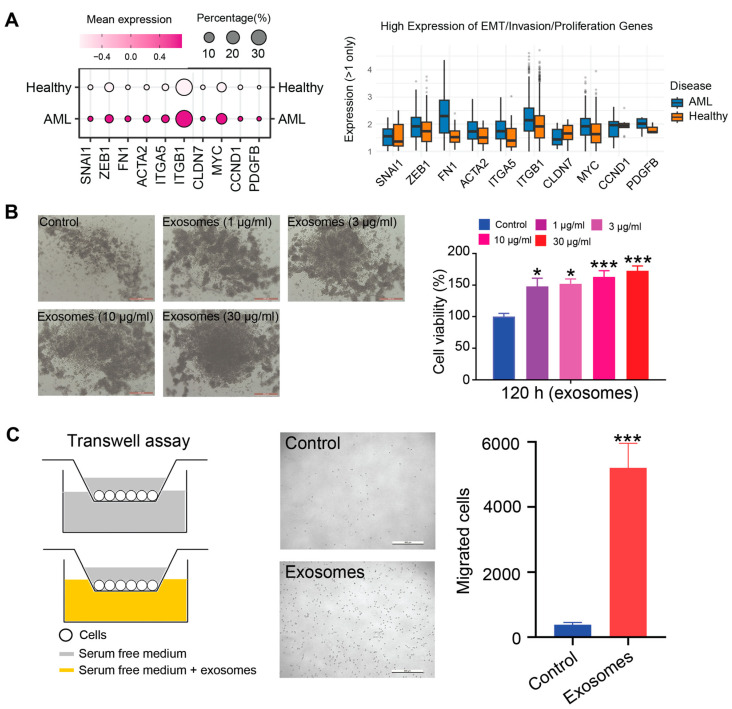
AML-exos affects proliferation and migration of Thp-1 cells. (**A**) Expression of cell migration, invasion, and proliferation markers between AML patients and healthy donors. (**B**) Representative images of Thp-1 cells after 120 h with different doses of AML-exos treatment (**left**). Scale bar = 500 μm. Cell viability of Thp-1 cells 120 h after culture with different doses of AML-exos measured by Cell Counting Kit-8 assay (**right**). * *p* < 0.05, *** *p* < 0.001. Error bars, s.e.m. One-way ANOVA. (**C**) The transwell assay model for studying the migration of Thp-1 cells after treatment with 30 μg/mL AML-exos (**left**). Representative images of Thp-1 cells after 18 h of transwell migration (**middle**). Scale bar = 500 μm. Migrated cells collected from the bottom wells were quantified by hemocytometry (**right**). *** *p* < 0.001. Error bars, s.e.m. Student *t*-test.

Several studies have implicated tumor-derived exosomes in the regulation of malignant behaviors such as proliferation and metastatic dissemination [30,31]. Based on the single-cell RNA sequencing data, we found a significant upregulation of genes associated with cellular invasion, motility, and expansion in AML patients compared to healthy donors, indicating that exosome-mediated signaling may play a pivotal role in reinforcing leukemic cell aggressiveness and disease progression (Figure 4A). To further substantiate these findings, we investigated the functional effects of AML-derived exosomes (AML-exos) on Thp-1 cells. Treatment of Thp-1 cells with increasing concentrations of AML-exos (0, 1, 3, 10, and 30 μg/mL) over a 120 h period resulted in a marked, dose-dependent enhancement of cell proliferation, as measured by CCK-8 assays (Figure 4B). Based on these results, 30 μg/mL was selected for subsequent analyses. In parallel, Transwell migration assays revealed a significant increase in Thp-1 cell motility following AML-exos exposure compared to untreated controls (Figure 4C). Collectively, these data indicate that AML-exos actively promote leukemic cell proliferation, migration, and invasive potential.

### 3.4. Exosomal TGF-β Promotes AML Cell Proliferation and Migration

**Figure 5:**
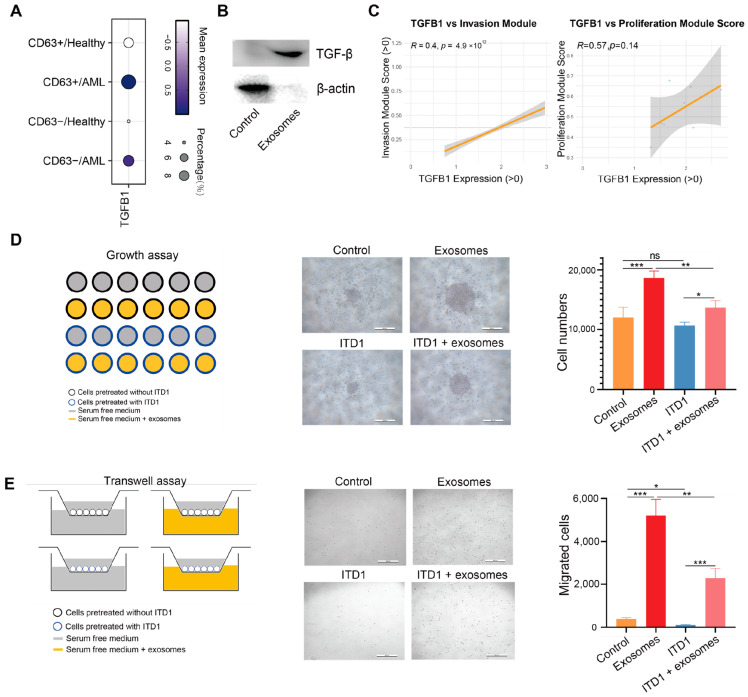
TGF-β carried by AML-exos accelerates proliferation and migration in Thp-1 cells. (**A**) Expression of TGFB1 in different groups. (**B**) The expression of TGF-β in the Thp-1 cell lysate (control), or AML-exos (Exosomes) was detected by Western blotting. (**C**) Pearson correlation of TGFB1 with invasion module or proliferation module in AML patients. (**D**) Growth assay of Thp-1 cells treated with AML-exos (30 μg/mL, 48 h) with or without the TGF-β receptor inhibitor ITD1 (5 μM). Scale bar = 500 μm. * *p* < 0.05, ** *p* < 0.01, *** *p* < 0.001 and ns *p* > 0.05 indicate no statistical significance. Error bars, s.e.m. One-way ANOVA. (**E**) Transwell assays were used to assess Thp-1 cell migration after AML-exos (30 μg/mL) treatment with or without TGF-β receptor inhibitor ITD1 (5 μM). Images were captured after 18 h of migration. Scale bar = 500 μm. * *p* < 0.05, ** *p* < 0.01, *** *p* < 0.001. Error bars, s.e.m. One-way ANOVA.

TGF-β, a key protein in tumor exosomes, is commonly elevated in cancer patients [32]. In the context of AML, TGF-β has been implicated in promoting leukemic cell survival, self-renewal, and immune evasion by reshaping the bone marrow microenvironment [33]. TGF-β signaling is often hyper-activated in AML, contributing to disease progression and resistance to therapy [34]. TGF-β is known to regulate a broad spectrum of cellular processes through its receptors [35]. Analysis of single-cell RNA sequencing data further supports this, revealing increased TGFB1 expression in AML samples compared to healthy controls (Figure 5A). Notably, TGFB1 expression was also elevated in CD63^+^ cell populations, suggesting a potential link between TGF-β and exosome-enriched compartments. Consistent with this observation, Western blotting showed TGF-β was significantly enriched in AML-exos treated group compared to control Thp-1 cells (Figure 5B). TGF-β has been found in various exosomes, impacting recipient cell growth and movement [36,37]. Additionally, Pearson correlation analysis revealed that in AML patients, TGFB1 expression positively correlates with the expression of genes associated with cell invasion and proliferation (Figure 5C). To further validate the functional role of TGF-β in AML-exos, we employed ITD1, a selective TGF-β receptor inhibitor. Treatment with ITD1 significantly attenuated the AML-exos-induced proliferation and migration of Thp-1 cells (Figure 5D,E), underscoring the critical contribution of exosomal TGF-β to leukemic cell behavior.

### 3.5. Exosomal TGF-β Induces ERK1/2 and Smad2/3–MMP2 Signaling and Suppresses Apoptosis

**Figure 6:**
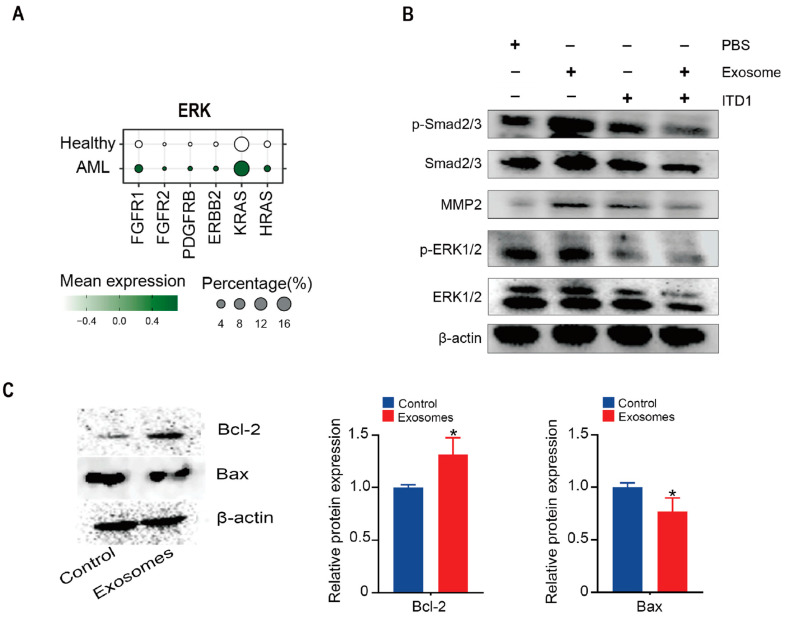
Exosomal TGF-β induces ERK1/2 and Smad2/3–MMP2 signaling. (**A**) Expression of genes associated with ERK in different groups. (**B**) The expression of TGF-β in the Thp-1 cell lysate (Control), or AML-exos (Exosomes) was detected by Western blotting. (**C**) Pearson correlation of TGFB1 with invasion module or proliferation module in AML patients. * *p* < 0.05, Error bars, s.e.m. Student *t*-test.

Aberrant activation of the ERK signaling pathway and suppression of apoptosis are well-documented features of AML, promoting leukemic cell proliferation and survival [38]. TGF-β signaling, frequently dysregulated in the AML microenvironment, has been shown to modulate both apoptosis and ERK activity through canonical and non-canonical pathways [39]. To further explore the downstream signaling of TGF-β in this context, we analyzed single-cell RNA-seq data and observed an upregulation of ERK-related genes in AML patients (Figure 6A). Treatment of Thp-1 cells with AML-exos significantly increased phosphorylation of ERK1/2 and Smad2/3 (Figure 6B), effects that were attenuated by the TGF-β receptor inhibitor ITD1. Given that Smad2/3 activation is associated with the expression of MMP2, a key mediator of cancer metastasis [40,41], we examined MMP2 levels and found that AML-exos upregulated MMP2 expression in Thp-1 cells, which was reversed by ITD1 (Figure 6B). These results suggest that TGF-β carried by AML-exos activates both the Smad2/3–MMP2 and ERK1/2 pathways in AML cells. Moreover, the expression of BCL-2 was increased, while BAX decreased, indicating that AML-exos promote an anti-apoptotic phenotype in Thp-1 cells (Figure 6C). These findings highlight a dual role of AML-exos in enhancing leukemic cell invasiveness and survival via TGF-β–mediated signaling. Targeting TGF-β-dependent ERK and Smad pathways may thus offer a promising strategy to counteract AML progression and exosome-driven malignancy.

### 3.6. The Impact of AML-Exos on Immune Modulation

**Figure 7:**
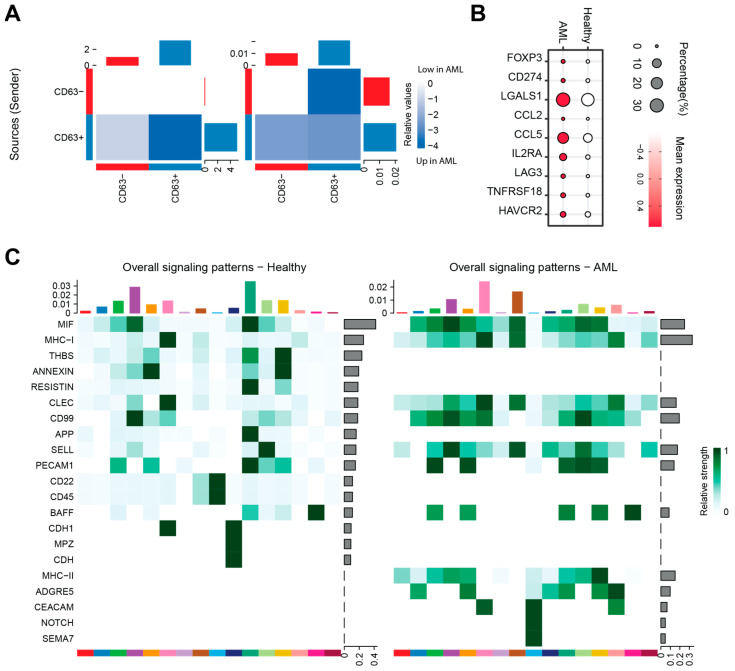
The function of AML-exos in immune modulation. (**A**) Heatmap of potential differential number and strength of cell–cell interaction. (**B**) The expression of immune modulation markers between AML patients and healthy donors. (**C**) Overall signaling patterns in healthy donors and AML patients. Different color indicates different celltypes.

We applied CellChat to investigate differences in potential cell–cell communication between AML and healthy groups, categorizing cells based on CD63 expression to distinguish exosome-activated (CD63^+^) and non-activated (CD63^−^) populations [42]. Our analysis revealed that both the number and strength of interactions were significantly increased in the AML group compared to healthy controls (Figure 7A), suggesting that exosome-mediated mechanisms enhance intercellular signaling. The expression of markers such as FOXP3, CD274, LGALS1, CCL2, CCL5, IL2RA, LAG3, TNFRSF18, and HAVCR2 were upregulated in the AML group, indicating that immune modulation processes were activated, potentially influencing T cell activation, differentiation, migration, and checkpoint signaling, and thereby reshaping the immune landscape within the leukemic microenvironment (Figure 7B).

Further pathway analysis showed significant upregulation of key immune-related signaling routes in AML patients, including CD99, SELL (L-selectin), MHC class I and II, Semaphorin (SEMA), NOTCH, and CEACAM (Figure 7C). These pathways are crucial for immune regulation in cancers: MHC-I and MHC-II facilitate antigen presentation to cytotoxic and helper T cells, respectively [43]; SELL and CD99 mediate leukocyte adhesion and trafficking, promoting immune cell migration [44]; Semaphorins modulate immune cell activation and movement beyond their classical roles in axon guidance [45]; NOTCH signaling influences immune cell differentiation and checkpoint regulation [46]; and CEACAM molecules act as immune checkpoints that may support immune evasion by cancer cells [47]. Notably, these pathways were predominantly enriched in CD63^+^ populations, supporting the hypothesis that AML-derived exosomes amplify immunomodulatory communication within the bone marrow microenvironment, potentially contributing to leukemic progression. These changes in surface marker expression further support the role of AML-derived exosomes in reshaping immune cell composition and function, thereby promoting an environment conducive to leukemia progression and immune evasion.

## 4. Discussion

AML stands as one of the most prevalent and lethal hematopoietic cancers, marked by high rates of incidence and recurrence. Investigating the molecular pathways involved in AML progression is therefore crucial. Exosomes, which are small vesicles released by tumor cells, play a pivotal role in cellular communication within tumors by transferring biologically active molecules to recipient cells [48]. The functional significance of tumor-derived exosomes in promoting tumor growth, facilitating metastasis, and affecting treatment response has become increasingly apparent [49]. Additionally, exosomes from leukemic blasts have been shown to inhibit hematopoiesis in AML [50]. Plasma-derived exosomes have also emerged as promising therapeutic targets for managing minimal residual disease in AML [51], underscoring the role of exosomes in AML progression.

Studies have suggested that exosomes originating from tumors play roles in various aspects of cancer progression, such as organ-specific metastasis [52], formation of new blood vessels [53], or evasion of immune responses [54]. Nonetheless, limited research has investigated how tumor cells respond when exposed to these exosomes. Importantly, our analysis reveals a clear correspondence between the molecular profiles of AML-derived exosomes and their cells of origin. Key transcriptional programs active in AML blasts, including those related to transcription, translation, and metabolism, were also enriched in exosomes. Using both in vivo and in vitro assays, and integrating RNA, protein, and cellular-level evidence, we comprehensively validated the biological functions of AML exosomes. These findings highlight exosomes as faithful reflections of the tumor state and support their potential as liquid biopsy biomarkers in AML.

Exosomes, as carriers of information, are hypothesized to transport specific molecules that play a role in AML progression. TGF-β, a versatile cytokine, exerts distinct effects depending on the cellular environment, acting as a growth inhibitor in early cancer stages and as a promoter in advanced malignancies. Beyond the traditional Smad pathway, TGF-β is also involved in non-Smad signaling, including the ERK pathway, which is crucial for cancer cell proliferation and survival. In this study, we observed that AML-exos contained TGF-β, and blocking TGF-β signaling pathways hindered the AML-exos-induced rise in cell proliferation and migration. Research has shown elevated TGF-β levels in the bone marrow, serum, and plasma of patients with bone marrow fibrosis or hairy leukemia [55,56]. Several studies highlight the TGF-β signaling pathway as significant in leukemia progression, specifically in maintaining leukemia-initiating cells through FOXO localization control [57,58]. Additionally, TGF-β signaling facilitates the recruitment and mobilization of leukemia stem cells into peripheral blood circulation [59]. Smad2/3 activation typically reflects TGF-β signaling, which is known to have dual roles in cancer [18]. In leukemic cells, sustained activation of Smad2/3 drives transcriptional programs that favor disease progression. Phosphorylated Smad2/3 can dysregulate the expression of cell cycle regulators such as p21 and c-MYC, promoting the self-renewal and survival of leukemic stem cells while inhibiting their differentiation [60]. Meanwhile, ERK1/2, a key component of the MAPK signaling cascade, is frequently upregulated in AML and contributes to uncontrolled proliferation, resistance to apoptosis, and metabolic reprogramming of leukemic cells [61]. The concurrent activation of both Smad2/3 and ERK1/2 pathways suggests a synergistic mechanism in AML. This dual signaling can promote a more aggressive leukemic phenotype by integrating external cues (e.g., from the bone marrow microenvironment or exosomes) with internal oncogenic programs. The crosstalk between these two pathways may create a feedback loop that sustains a resistant, stem-like cellular state, facilitating leukemic progression even under therapeutic pressure. This dual-pathway activation thus represents a critical mechanism of therapeutic failure and highlights the need for combinatorial strategies to effectively target LSCs in AML.

Moreover, our study reveals that AML-derived exosomes significantly upregulate multiple immune-related signaling pathways, including CD99, SELL (L-selectin), MHC class I and II, Semaphorin, NOTCH, and CEACAM. These pathways play essential roles in antigen presentation, immune cell trafficking, activation, and immune checkpoint regulation, and are predominantly enriched in CD63^+^ exosome populations. This suggests that AML exosomes actively reshape the bone marrow immune microenvironment to favor leukemic progression and immune evasion.

Interestingly, many of these immune pathways exhibit functional and mechanistic parallels to EMT which is associated with increased cell motility, invasion, immune suppression, and microenvironment remodeling. For example, SELL and CD99 facilitate cell adhesion and migration akin to EMT-driven invasiveness; dysregulated MHC pathways contribute to immune escape similar to EMT-associated immunosuppression; Semaphorins regulate cytoskelet al dynamics and plasticity central to EMT morphology; NOTCH signaling promotes mesenchymal traits and stemness; and CEACAMs act as immune checkpoints facilitating immune evasion. The activation of these pathways downstream of TGF-β, converging on Smad2/3 and ERK1/2, further supports that AML-derived exosomes induce EMT-like features despite the absence of classical EMT in hematologic malignancies. Therefore, our data suggests that exosome-mediated signaling drives a functional EMT analog in AML, promoting leukemic cell aggressiveness and immune modulation within the bone marrow niche. Nonetheless, further functional studies are required to validate these pathways’ precise roles and their contributions to AML pathogenesis.

While our study highlights the exosome-mediated mechanism in AML, this phenomenon is not unique to AML and has also been reported in various other hematologic malignancies. For example, in chronic lymphocytic leukemia (CLL), exosomes derived from leukemic cells have been shown to modulate the tumor microenvironment by promoting stromal cell activation, immune evasion, and drug resistance [62]. Similarly, in multiple myeloma, tumor-derived exosomes carry cytokines, microRNAs, and surface molecules that support angiogenesis, osteoclast activation, and the suppression of T cell responses [63]. In lymphoma, exosomes have been implicated in remodeling the extracellular matrix and facilitating metastatic spread [64]. These findings suggest that the ability of exosomes to mediate intercellular communication and support malignant progression is a conserved mechanism across multiple hematologic cancers. However, the specific molecular cargo and functional outcomes of exosomal signaling may vary depending on the disease context. In AML, our study provides further evidence that exosomes not only reflect the leukemic transcriptome but also actively contribute to proliferation and signaling activation, reinforcing their potential as both biomarkers and therapeutic targets.

The mechanism by which TGF-β is selectively sorted into exosomes remains an area of active investigation, but several hypotheses and emerging studies offer insight. One proposed mechanism involves the interaction of TGF-β with the ESCRT (endosomal sorting complexes required for transport) machinery and associated adaptor proteins, which facilitate the inclusion of specific cargo into intraluminal vesicles (ILVs) during exosome biogenesis [65]. Functionally, exosome-bound TGF-β differs from its soluble form in terms of bioavailability, stability, and receptor engagement [66]. Soluble TGF-β is diffusible and rapidly degraded in the extracellular space, whereas exosomal TGF-β is membrane-associated and protected from enzymatic cleavage, enabling prolonged signaling activity and localized delivery [67]. Furthermore, exosomal TGF-β may preferentially interact with receptors on specific target cells via ligand clustering or co-delivery with other co-stimulatory molecules, potentially enhancing signaling potency [68]. In the context of AML, such targeted and sustained delivery could facilitate more robust Smad2/3 activation, promoting leukemic stemness and immune modulation within the bone marrow niche. These observations underscore the functional importance of delivery mode and support the notion that exosomal TGF-β is not merely a surrogate for the soluble form, but a distinct and biologically potent signaling modality.

Recent studies indicate that exosomes may serve as effective carriers for delivering anticancer drugs [69]. Additionally, some research highlights exosome inhibitors, such as GW4869, as potential therapeutic strategies for various conditions, including Duchenne muscular dystrophy [70] and heart dysfunction [71]. Our findings propose that targeting AML-exos could be a viable approach for therapy. However, exosomes and their key marker proteins are involved in essential biological processes, like fertilization and aging [72,73], suggesting that exosome inhibition in therapy could result in significant side effects. Given their ability to mirror the transcriptional and functional landscape of leukemic cells, exosomes hold promises for use in liquid biopsy to monitor disease progression, detect minimal residual disease, and predict therapeutic response. Furthermore, targeting exosome-mediated signaling may offer novel therapeutic strategies aimed at disrupting leukemic communication networks and overcoming resistance. These findings lay the groundwork for future translational efforts to integrate exosome profiling into the clinical management of AML.

Despite the strength of our integrative approach, several limitations of this study should be acknowledged. First, although we demonstrated the functional and molecular alignment between AML-derived exosomes and their cells of origin, the findings are primarily based on ex vivo and in vitro systems, which may not fully capture the complexity of the in vivo bone marrow microenvironment. Second, while we focused on a subset of key signaling pathways such as Smad2/3 and ERK1/2, other potentially relevant exosomal components and signaling mechanisms remain unexplored. Third, the heterogeneity of AML subtypes and inter-patient variability were not fully addressed, and future studies using larger, clinically diverse cohorts are needed to validate the generalizability of our findings.

## 5. Conclusions

In summary, our study highlights the significant role of exosomes derived from AML cells in enhancing the proliferation, migration, and apoptosis resistance of AML cells via the TGF-β signaling pathway. The ability of AML-exos to activate key pathways, specifically the Smad2/3-MMP2 and ERK1/2 pathways, suggests their influential role in AML progression and metastatic potential. These insights underline the relevance of TGF-β in exosome-mediated AML cell communication, opening prospects for targeted therapeutic strategies. Given the impact of TGF-β carried by AML-exos on AML cell behaviors, future research should consider designing therapies to selectively inhibit the influence of exosomal TGF-β or to modulate it signaling pathways. Additionally, exploring other potential factors within AML-exos could unveil further molecular mechanisms contributing to AML advancement. Combining this approach with an investigation of exosome interactions with other cell types in the AML microenvironment may provide a comprehensive understanding of exosome-driven AML dynamics. These efforts could lead to innovative and more precise therapies aimed at limiting AML cell survival and metastasis, thereby improving clinical outcomes in AML patients.

## Data Availability

Thp-1 human acute myeloid leukemia cell line was acquired from the American 75 Type Culture Collection (ATCC TIB-202). The original contributions presented in this study are included in the article.
